# The effect of biceps tenotomy on humeral migration and clinical outcomes in arthroscopic rotator cuff repair

**DOI:** 10.1186/s40634-022-00550-3

**Published:** 2022-11-30

**Authors:** Burak Çakar, Ahmet Güney, Betül Güney, Erdal Uzun, Hazım Sekban

**Affiliations:** 1 Orthopedics and Traumatology Department of Erciyes University Medical Faculty Hospital, Kayseri, Turkey; 2 Orthopedics and Traumatology Department of Erciyes University Medical Faculty Hospital, Kayseri, Turkey; 3 Erciyes University Halil Bayraktar Vocational School, Kayseri, Turkey; 4 Kayseri City Hospital, Kayseri, Turkey

**Keywords:** Long head, Biceps, Tenotomy, Rotator cuff tears, Arthroscopy, Humeral migration

## Abstract

**Purpose:**

To evaluate the effect of biceps tenotomy on humeral migration and clinical outcomes in patients who underwent arthroscopic rotator cuff (RC) repair.

**Methods:**

This is a retrospective study of 60 patients who underwent arthroscopic RC repair. Patients were divided into two groups, whether they underwent concomitant biceps tenotomy or not. The group underwent concomitant biceps tenotomy, tenotomy ( +), or not, tenotomy (-). Clinical and functional outcomes were performed using the American Shoulder and Elbow Surgeons (ASES), the University of California-Los Angeles (UCLA) scoring system.

Radiological evaluation was performed in X-rays and magnetic resonance imaging (MRI), measuring the acromiohumeral distance (AHD), humeral migration (HM) and upper migration index (UMI).

**Results:**

There was no significant difference between the groups in terms of patient characteristics. The follow-up period was 30.9 ± 8.7 months in the tenotomy ( +) group and 34.9 ± 8.2 months in the tenotomy (-) group with no significant difference. Postoperative ASES score improved significantly in the tenotomy ( +) group compared to the tenotomy (-) group (91.2 ± 4.7, 80.8 ± 18.7, respectively, *p* = 0.005). There was a significant difference in postoperative AHD, HM and UMI values (MRI; *p* = 0.003, *p* = 0.017, *p* = 0.025; X-ray; *p* = 0.049, *p* = 0.002, *p* = 0.010, respectively). The post–pre difference increase of AHD [MRI for tenotomy( +): 0.14 ± 0.86 and tenotomy(-): 0.91 ± 0.85, *p* = 0.001; X-ray for tenotomy( +): 0.61 ± 0.43 and tenotomy(-): 1.12 ± 0.7, *p* = 0.001] and UMI [MRI for tenotomy( +): 0.005 ± 0.05 and tenotomy(-): 0.04 ± 0.06, *p* = 0.006; X-ray for tenotomy( +): 0.01 ± .064 and tenotomy(-): 0.12 ± 0.37, *p* = 0.110] values were higher in the tenotomy (-) group compared to the tenotomy ( +) group while HM values decreased more in the tenotomy (-) group. [MRI for tenotomy ( +): -0.19 ± 1.07 and tenotomy (-): -0.79 ± 1.52, *p* = 0.079; X-ray for tenotomy ( +): -0.27 ± 0.54 and tenotomy (-): -1.006 ± 1.83, *p* = 0.040].

**Conclusion:**

After short-term follow-up, the humeral head was positioned higher in patients who underwent LHBT tenotomy compared to patients without tenotomy. However, it seems to affect clinical outcomes during this period positively.

**Level of Evidence:**

Level 3

## Introduction

Long head of biceps tendon (LHBT) lesions have increasingly been associated with RC tears as a major cause of shoulder pain. However the management of long head of biceps tendon (LHBT) pathology in the context of rotator cuff repair is still controversial [4]. The higher likelihood of LHBT pathology, with the increasing size of RC tears, may be a consequence of its functioning as a suppressor of the humeral head [1]. A study showed that any RC tear compromises the dynamic stabilizers of the shoulder and significantly leads to superior humeral migration [21]. Subacromial impingement may also contribute to LHBT pathology in the presence of an RC tear [23]. A recent study found that biceps pathology reduced AHD. They also showed that this decrease was greater in patients with RC tears [19].

Superior migration of the humeral head after RC tear was first described by Golding in 1962 [7]. Acromiohumeral distance (AHD) measurement on direct radiographs has been defined as a helpful method in evaluating RC disorders. Proximal humeral migration has been observed both clinically and experimentally in RC deficient shoulders [29]. A recent study showed that there is a high correlation between the different parameters of superior humeral migration when measured with either radiography or MR imaging [16].

The role of LHBT in the shoulder is controversial, partly because of the relatively few studies on the subject. LHBT has been described as suppressing the humeral head [12]. Warner and McMahon [27] reported superior humerus displacement during arm abduction in isolated LHBT tears in shoulders without RC tears; this is indirect evidence that the biceps acts as a suppressor. On the other hand LHBT tenotomy provides pain relief, minimal residual symptoms, and satisfactory results when performed with RC repair [1, 15, 26, 28].

The present study aimed to investigate the effect of biceps tenotomy on humeral migration and clinical outcomes in patients with RC repair. The hypothesis in the study was that concurrent biceps tenotomy would change humeral migration and clinical outcomes in patients who underwent RC tear repair.

## Materials and methods

### Patients

**Fig. 1:**
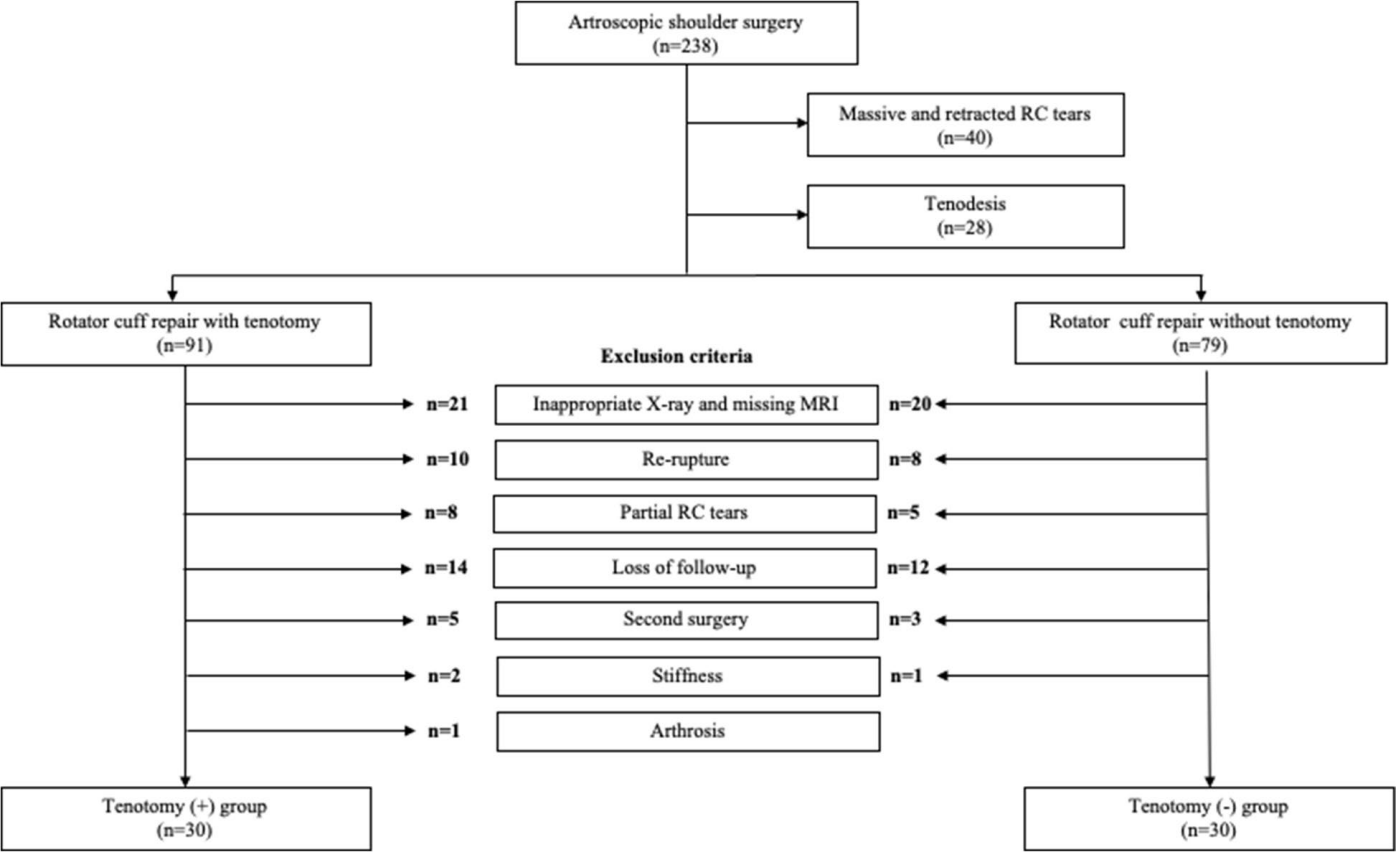
Flow diagram

This study was approved by the institutional review board of Erciyes University with decision number 2020/317 and informed consent was obtained from the patients. This is a retrospective study of prospectively collected data on 238 patients who underwent arthroscopic shoulder surgery between 2014 and 2019 in our clinic. Patient data were obtained from the patient files and images in the hospital document registration system. Inclusion criteria were at least a 24 months follow-up period, having standard true anterior–posterior (AP) radiography and appropriate quality magnetic resonance imaging (MRI) images, tear in the supraspinatus and/or infraspinatus tendons, grade 1 or 2 retraction in the Patte classification [17], fatty degeneration grade I or II according to Goutallier [8], tears smaller than 5 cm and RC repairs with double-row repair technique. The exclusion criteria were surgical history of the same shoulder, detection of re-rupture, partial RC tears, massive and retracted RC tears, shoulder instability, shoulder stiffness, application of biceps tenodesis, not attending regular follow-ups, not following the rehabilitation program, and shoulder joint osteoarthritis. Patient selection criteria and exclusion criteria are shown in Fig. 1.

The cuff was evaluated preoperatively by MRI and arthroscopically. The tear size was measured with the help of an arthroscopic probe and was also assessed for repairability. Biceps tenotomy was performed in the presence of tendon abrasion, partial tear, or advanced tendonitis during the surgery. The five stage assessment system developed by Sugaya et al. [22] was used to evaluate tendon repair from MRI images and patients with type 1–3 were included in the study. Evaluation of tendon healing was performed by an investigator that was blinded to patient data. Biceps tenotomy was performed on 30 patients who met the inclusion criteria, and these were called the tenotomy ( +) group. 30 patients who did not undergo tenotomy were called the tenotomy (-) group. There was no significant difference between the groups in terms of patient characteristics, follow-up time and tears characteristics. The demographic data of the patients are shown in Table 1. Intraoperative tear characteristics are shown in Table 2.

**Table 1:** The demographic data of the patients

	**Total (*n* = 60)**	**Tenotomy( +) (*n* = 30)**	**Tenotomy(-) (*n* = 30)**	***P* value**
**Age (year), Mean ± Sd**	57.38 ± 7.46	56.80 ± 6.62	57.97 ± 8.28	0.54
**Follow up time (mount) Mean ± Sd**	32.95 ± 8.65	30.93 ± 8.73	34.97 ± 8.22	0.07
**Gender, n (%)**				0.06
Female	38 (63.3)	23 (76.6)	15 (50)	
Male	22 (36.7)	7 (23.4)	15 (50)	
**Affected side, n (%)**				0.99
Right	41 (68.3)	21 (70)	20 (66.7)	
Left	19 (31.7)	9 (30)	10 (33.3)	
**Dominant side, n (%)**				0.70
Right	52 (86.7)	27 (90)	25 (83.3)	
Left	8 (13.3)	3 (10)	5 (16.7)	
**Comorbidity, n (%)**				0.99
No	23 (38.3)	11 (36.6)	12 (40)	
Yes	37 (61.7)	19 (63.4)	18 (60)	
**Actively working, n (%)**				0.170
No	40 (66.7)	23 (76.7)	17 (56.7)	
Yes	20 (33.3)	7 (23.3)	13 (43.3)	

Diabetes mellitus, hypertension, goiter, asthma, chronic obstructive pulmonary disease, coronary artery disease

**Table 2:** Intraoperative tear characteristics

	**Tenotomy ( +) (*n* = 30)**	**Tenotomy (-) (*n* = 30)**	**p**
**Number of tendon torn**			1.000
1	26	27	
≥ 2	4	3	
**Tear size**			0.559
< 2 cm	21	23	
≥ 2-5 cm	9	7	
**Fatty infiltration**			1.000
Grade 0	9	8	
Grade 1	17	17	
Grade 2	4	5	
**Tendon retraction**			0.542
Grade 1	22	24	
Grade 2	8	6	
**Tendon torn**			
Supraspinatus	28	30	0.492
Subscapularis	2	1	1.000
İnfrasupinatus	4	2	0.671
Teres minör	0	0	-

### Surgical technique

All patients were operated on in the beach chair position by a single senior orthopedic surgeon, using the same surgical technique with interscalene block and/or general anesthesia. An arthroscope was introduced to the glenohumeral joint through a posterior viewing portal, and intraarticular pathologies were scrutinized, especially the biceps status. Tenotomy with the aid of arthroscopic cautery was applied to patients with pathology in evaluating biceps tendon. Repair or debridement was performed in patients with subscapularis tear. After intraarticular evaluation, the subacromial space was evaluated. After debridement, the RC tear was repaired with a double-row repair technique using an appropriate number of suture anchors according to tear size.

### Rehabilitation

The same rehabilitation program was applied in both groups after surgery. Abduction orthosis was applied to the patients for six weeks postoperatively. Shoulder immobilization was applied for the first three weeks, and elbow and wrist exercises were recommended during this period. After the 3rd week, only passive range of motion (ROM) exercises were performed until the sixth week while waiting for the repair tissue to heal. Active ROM exercises were initiated on the 7th-9th weeks, with limited ROM; minimum resistance exercises were allowed on the 10th-12th weeks. Strengthening exercises against resistance were started between 12. and 24. weeks. It was aimed to provide dynamic functional stability by strengthening the joint. However, after the 24th week, the patients were allowed to return to sports.

### Assessment

Functional outcomes were evaluated using the American Shoulder and Elbow Surgeons (ASES) and the University of California-Los Angeles (UCLA) scoring systems, which were recorded during the preoperative preparation and postoperative at least after two years controls of the patients. In addition, the joint range of motion (ROM) of the patients was evaluated by measuring the active shoulder joint elevation. Active shoulder ROM was measured with a plastic goniometer for elevation with the patient in a standing position. The radiological evaluation was made from the preoperative and postoperative MRI and standard true AP radiographs in the hospital system of the patients. Postoperative images were used which taken after at least 2 years of follow-up. Radiological outcomes were evaluated by measuring acromiohumeral distance (AHD) [29], humeral migration (HM) [2, 18], upper migration index (UMI) [9]. A standard protocol AP radiograph was taken of all patients with the patient in the supine position, slightly turned to the image side (30°), and the arm in neutral rotation with the palm facing forward. The film-focus distance was measured at 120 cm, and a 15° craniocaudal tilt was used to project the acromion perpendicular undersurface. This created a true AP projection 90° toward the glenohumeral joint. To standardize the measurements, a radio-opaque material of known size was placed in the cassette.

**Fig. 2:**
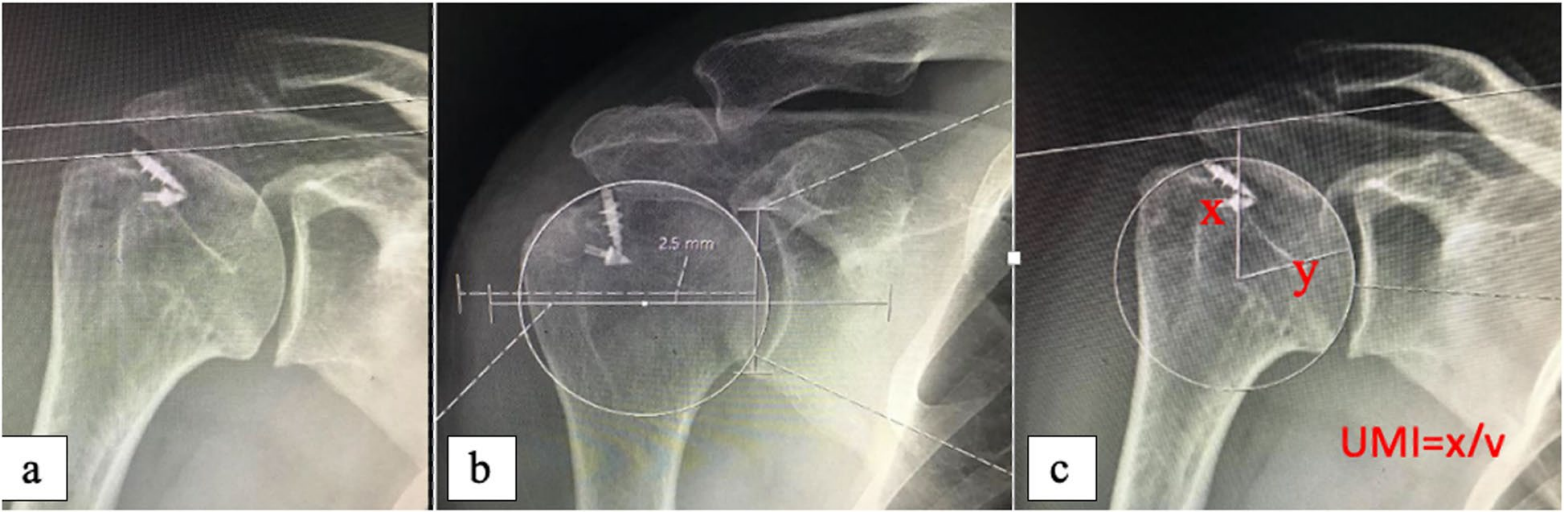
Radiological measurements at X-ray. **a** Acromiohumeral distance measurements. **b** Humeral migration measurements, **c** Upper migration index formula and measurements

**Fig. 3:**
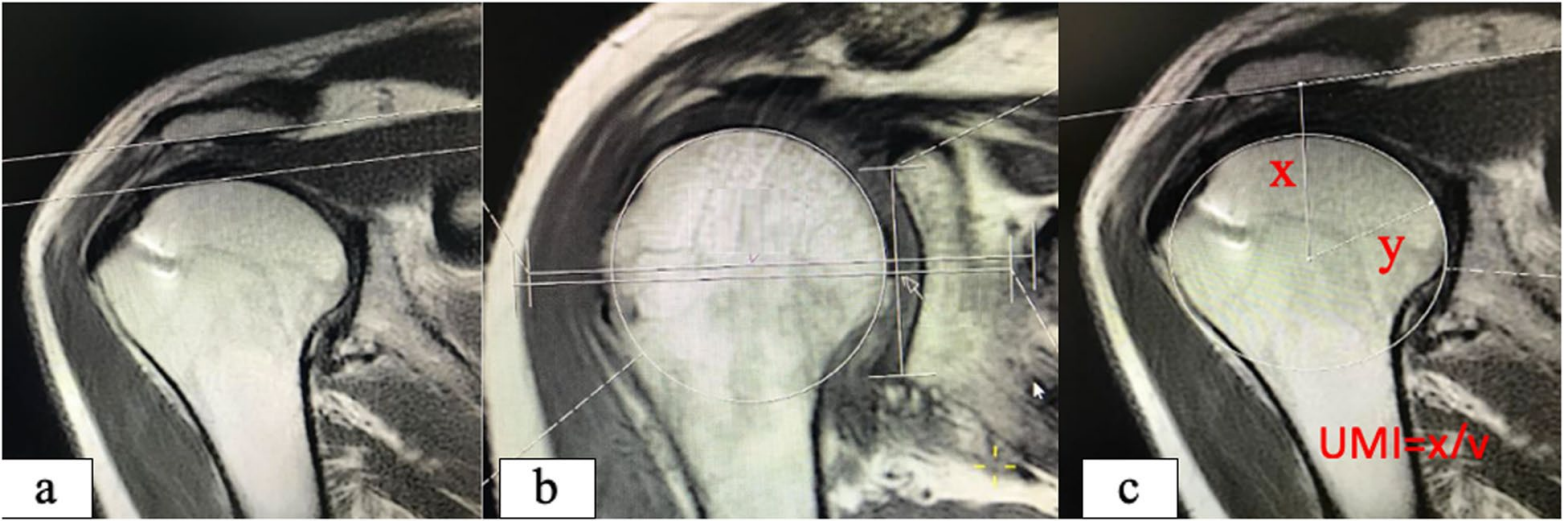
Radiological measurements at MRI. **a** Acromiohumeral distance measurements, **b** Humeral migration measurements, **c** Upper migration index formula and measurements. **a** AHD was defined as the distance between the lowest border of the acromion and the humeral head's highest point. **b** HM value, the humeral head center, was determined by drawing a tangent circle to the humeral head's boundaries. Then, the glenoid fossa was determined by the line drawn between the superior and inferior most edges of the glenoid, and then the center of the glenoid fossa was marked on this line. A second line was drawn from the center of the humeral head perpendicular to the glenoid fossa line, and the intersection point was marked. The distance between the intersection point and the center of the glenoid fossa was recorded as the HM. If the center of the glenoid fossa was inferior to the intersection point, the HM was recorded as a negative value. If it was superior, then the HM took a positive value. **c** The UMI, where the distance between the center of the humeral head to the acromion’s undersurface was divided by the humeral head’s radius, as described by Hirooka et all

Each parameter of superior humeral migration was measured twice with a three-week interval by two blinded investigators separately and randomly to calculate interrater and intrarater reliability. The inter-observer agreement (ranged between 0.90 and 0.95) and the intra-observer agreement (ranged between 0.88 and 0.92) were very high. The mean values of the measured variables were used for analysis. Radiological measurements are shown in Figs. 2 and 3.

### Statistical analysis

The data analysis was made with the SPSS Statistics version 26.0 (IBM Inc., Chicago, IL, USA) program. Sample size calculation was based on both MR and X-ray AHD measurements. When 30 patients were collected in both groups and the type 1 error was determined as 5%, the power of the study was calculated as over 90%. The compliance of quantitative data to normal distribution was examined using the Kolmogorov Smirnov test. Quantitative data conforming to normal distribution are defined as x ± SD. The difference between groups was analyzed using Student's t-test. In the quantitative data, the Mann–Whitney U test was used for data that did not conform to a normal distribution. Qualitative data were defined as %. The difference between the groups was analyzed using the Chi-square test (× 2). The intraclass correlation coefficients were calculated to evaluate the intra-observer and inter-observer agreement. The level of significance was taken as 0.05.

## Results

### Clinical outcomes

Intergroup comparison of functional outcomes is shown in Table 3. Preoperative ASES and UCLA scores did not significantly differ between the groups. Postoperative ASES and UCLA scores improved significantly in all patients than preoperative values (*p* = 0.001, *p* = 0.001, respectively). ASES scores were significantly higher in the tenotomy ( +) group compared to the tenotomy (-) group postoperatively (*p* = 0.005). However, there was no significant difference regarding the postoperative UCLA scores between the groups. There was no significant difference between the groups in terms of preoperative and postoperative active shoulder elevation. Three (10%) patients (two male, one female) who underwent tenotomy had the Popeye sign at the last follow-up control. No other early or late complication in the patient groups was observed.

**Table 3:** Pre- and postoperative functional outcomes of patients

	**Total (*n* = 60)**	**Tenotomy( +) (*n* = 30)**	**Tenotomy(-) (*n* = 30)**	***p* value**
	**Mean ± Sd**	**Mean ± Sd**	**Mean ± Sd**	
**ASES Score**				
Preop	39.46 ± 6.61	39.38 ± 6.51	39.54 ± 6.82	0.926
Postop	85.99 ± 14.48	91.16 ± 4.70	80.83 ± 18.69	**0.005**
Postop-preop difference	46.54 ± 16.76	51.78 ± 6.63	41.30 ± 21.7	**0.016**
*p* value	**0.001**	**0.001**	**0.001**	
**UCLA score**				
Preop	14.6 ± 3.5	14.37 ± 3.56	14.83 ± 3.48	0.610
Postop	30.6 ± 4.06	30.96 ± 2.41	30.23 ± 5.23	0.489
Postop-preop difference	16.0 ± 5.77	16.6 ± 4.53	15.4 ± 6.82	0.425
*p* value	**0.001**	**0.001**	**0.001**	
**Active elevation ROM (degree)**				
Preop	102.17 ± 8.45	100.23 ± 8.11	104.12 ± 8.46	0.075
Postop	164.63 ± 6.22	164.6 ± 6.40	164.66 ± 6.15	0.967
Postop-preop difference	62.45 ± 10.57	64.36 ± 10.66	60.54 ± 10.3	0.163
*p* value	**0.001**	**0.001**	**0.001**	

*ROM* Range of motion, *ASES* American Shoulder and Elbow Surgeons, *UCLA* The the University of California-Los Angeles

### Radiological outcomes

Pre- and postoperative radiological measurement values of the patients are shown in Table 4. There was no significant difference in AHD, HM and UMI values between groups in preoperative MRI and X-ray measurements. Postop-preop difference in MRI was significant in AHD, HM and UMI total (*p* = 0.001, *p* = 0.006, *p* = 0.001, respectively) and tenotomy (–) group (*p* = 0.001, *p* = 0.008, *p* = 0.001, respectively), while it was insignificant in tenotomy ( +) group.

**Table 4:** Pre- and postoperative radiological measurement values of the patients

	**Total (*n* = 60)**	**Tenotomy( +) (*n* = 30)**	**Tenotomy(-) (*n* = 30)**	***p* value**
	**Mean ± Sd**	**Mean ± Sd**	**Mean ± Sd**	
**MRI AHD (mm)**				
Preop	7.78 ± 0.9	7.70 ± 1.05	7.85 ± 0.73	0.524
Postop	8.30 ± 1.24	7.84 ± 1.18	8.76 ± 1.14	**0.003**
Postop-preop difference	0.52 ± 0.93	0.14 ± 0.86	0.91 ± 0.85	**0.001**
*p* value	**0.001**	0.392	**0.001**	
**MRI HM (mm)**				
Preop	0.36 ± 1.16	0.37 ± 1.17	0.33 ± 1.15	0.886
Postop	-0.13 ± 1.07	0.19 ± 0.94	-0.46 ± 1.10	**0.017**
Postop-preop difference	-0.49 ± 1.34	-0.19 ± 1.07	-0.79 ± 1.52	0.079
*p* value	**0.006**	0.350	**0.008**	
**MRI UMI**				
Preop	1.35 ± 0.08	1.36 ± 0.07	1.35 ± 0.10	0.675
Postop	1.38 ± 0.06	1.36 ± 0.03	1.39 ± 0.07	**0.025**
Postop-preop difference	0.02 ± 0.06	0.005 ± 0.05	0.04 ± 0.06	**0.006**
*p* value	**0.001**	0.622	**0.001**	
**X-ray AHD (mm)**				
Preop	7.52 ± 1.30	7.36 ± 1.40	7.68 ± 1.20	0.337
Postop	8.39 ± 1.68	7.97 ± 1.65	8.81 ± 1.63	**0.049**
Postop-preop difference	0.87 ± 0.63	0.61 ± 0.43	1.12 ± 0.70	**0.001**
*p* value	**0.001**	**0.001**	**0.001**	
**X-ray HM (mm)**				
Preop	0.59 ± 1.27	0.75 ± 1.38	0.44 ± 1.16	0.349
Postop	-0.04 ± 1.54	0.48 ± 1.52	-0.56 ± 1.39	**0.002**
Postop-preop difference	-0.64 ± 1.38	-0.27 ± 0.54	-1.006 ± 1.83	**0.040**
*p* value	**0.001**	**0.009**	**0.005**	
**X-ray UMI**				
Preop	1.31 ± 0.05	1.30 ± 0.03	1.32 ± 0.07	0.254
Postop	1.38 ± 0.27	1.32 ± 0.06	1.45 ± 0.37	**0.010**
Postop-preop difference	0.06 ± 0.27	0.01 ± .064	0.12 ± 0.37	0.110
*p* value	0.061	0.354	0.081	

*MRI* Magnetic Resonance Imagining, *AHD* Acromiohumeral distance, *HM* Humeral migration, *UMI* Upper migration index

Postop-preop difference AHD and HM values ​​were found to be statistically significant in total and between groups in X-ray measurements (total; *p* = 0.001, *p* = 0.001, tenotomy ( +); *p* = 0.001, *p* = 0.009, tenotomy (-); *p* = 0.001, *p* = 0.005, respectively), UMI values ​​were found to be insignificant in total and between groups.

In the tenotomy ( +) group, AHD values were found to be less increased, and HM was higher compared to the tenotomy (-) group. Besides, UMI values were significantly lower in the tenotomy ( +) group, depending on the decrease in AHD, compared to the tenotomy (-) group.

## Discussion

The most important finding of this study is that LHBT tenotomy applied together with RC repair affects the clinical results and superior migration of the humerus. While LHBT tenotomy has a positive effect on clinical results, it causes the humerus to displace upwards due to removing its suppressive effect on the humeral head.

In our study, it was shown that the humeral head was displaced downwards due to RC repair and the suppressing effect of RC on the humeral head. But the most crucial thing in this study was to evaluate the difference between the two groups.

In a recent study showed that RC tear causes humeral migration by affecting the dynamic stabilizers of the shoulder [21]. In addition, about imaging methods to evaluate the displacement of the humeral head in RC tears, they evaluated the benefits of parameters such as upward migration index, inferior glenohumeral distance, acromial index and critical shoulder angle other than AHD. AHD is accepted as a prognostic indicator that affects functional outcome [5, 13, 14, 20].

In this study, pre-and postoperative AHD values measured from MRI and X-ray images are consistent with other studies [3]. Postoperative AHD values seemed to increase due to RC repair. The main point to be considered was that the difference between groups is significant. Less increase in AHD values in the tenotomy ( +) group than the tenotomy (-) group indicated the depressing effect of LHBT on the humerus head. Besides, most biomechanical studies on the function of LHBT have been conducted on cadavers and focused on the impact of GH on joint stability, with controversial results [11]. In vivo biomechanical studies have shown the upward migration of the humeral head in the absence or non-stimulation of LHBT; thus, it has been concluded that it functions as a humeral head suppressor [12].

Hirooka et al. [9] described an alternative method to measure the upper migration of the humerus and expressed the AHD value as a UMI ratio. Van de Sande and Rozing [24, 25], demonstrating a high correlation between plain films and computed tomography scans, determined the UMI measurement accuracy on plain radiographs. In our study, we found similar results in measurements made from MRI and X-ray images. This is because AHD is narrower in the tenotomy ( +) group. However, while the postop-preop UMI difference was substantial in MRI measurements in the total and tenotomy (-) group, this difference was not marked in X-ray images, suggesting that MRI performed more sensitive measurements.

In the study of Çetinkaya et al. HM and AHD were evaluated in four different patient groups, each of which included 30 patients [2]. HM and AHD measurements were made for all patients. The correlation of the two measurements was examined, they showed that AHD and HM measurements had a high correlation in all patient groups. In the present study, HM values gave similar results as AHD and UMI values. The absence of LHBT causes the humeral head center to be positioned above the glenoid center in the tenotomy ( +) group. Another study showed that the movement of the head in RC torn shoulders approximates the movement of normal shoulders after the biceps contraction. This suggests that the active biceps contraction can compensate for the suppressing function of the RC [12]. From a functional perspective, the evidence for the LHBT being a humeral head depressor and glenohumeral stabilizer has been demonstrated in both in vitro biomechanical studies and in vivo EMG studies. However, it is also important to recognize that even if the biceps muscle is not activated, the LHBT in a passive state still contributes to glenohumeral joint stability through barrier effects of the soft tissue alone [6].

In the study, the effect of biceps tenotomy on clinical results was evaluated with ASES and UCLA scores. Studies have found that LHBT tenotomy provides pain relief and it was also observed that biceps tenotomy with RC repair yielded better results [15, 28].

In our study, after an average of 33 months of follow-up, postoperative ASES scores were found to be significantly higher in the tenotomy ( +) group. This difference was thought to be due to the rapid improvement of daily activities evaluated in the ASES score after tenotomy.

The Popeye deformity and cramping pain are two critical reasons for not choosing tenotomy. Qiang et al. [30] showed in their study that the Popeye sign was minimally noticeable after tenotomy. On the other hand, they reported the incidence of Popeye's sign as 9.1% after tenotomy. In this study, the Popeye sign was seen in three (two male, one female) patients (10%) who underwent tenotomy, and the patients did not complain about this condition. Our findings were consistent with the findings of the previous study [10, 18].

One of the limitations of the study is the short-term follow-up period. After a mean follow-up of 33 months, no pathology was observed in the GH joint secondary to humeral migration. After long-term follow-up, it should be investigated whether any pathology due to humeral migration would develop in the GH joint and how the patients would be affected clinically by humeral migration. Another limitation is that the study was retrospective and the number of patients in the group was small. Finally, tenotomy was applied to the patients with biceps pathology, but not to the healthy ones, so randomization could not be performed.

In the light of the findings, biceps tenotomy clinically gave better results in ASES scores. While LHBT tenotomy clinically provided more improvement in patients, radiologically, it was observed that it caused superior humeral migration. Despite this, it was thought that the patients' clinical results were not negatively affected since humeral migration did not cause severe narrowing in AHD after RC repair.

Tenotomy should be performed in elderly patients with LHBT pathology. They will show a significant improvement clinically. The long-term outcomes of biceps tenotomy in younger active patients are unknown. In these patients, other treatment alternatives should be considered. Studies evaluating the effect of biceps tenotomy on long-term clinical and radiological results after RC repair are needed.

## Conclusion

Long head of the biceps tenotomy combined with the RC repair affects clinical and radiological outcomes. The humeral head was positioned higher in the LHBT tenotomy group compared to the group without tenotomy. However, after short-term follow-up, it seems to have a positive effect on clinical outcomes in short-term.

## Abbreviations

AHD: Acromiohumeral Distance
AP: Anterior–posterior
ASES: American Shoulder and Elbow Surgeons
HM: Humeral Migration
LHBT: Long Head of Biceps Tendon
MRI: Magnetic Resonance Imagining
RC: Rotator Cuff
ROM: Range of Motion
UCLA: The University of California-Los Angeles
UMI: Upper Migration Index
